# Influence of Heat Exposure on Motor Control Performance and Learning as Well as Physiological Responses to Visuomotor Accuracy Tracking Task

**DOI:** 10.3390/ijerph191912328

**Published:** 2022-09-28

**Authors:** Mao Aoki, Yudai Yamazaki, Junto Otsuka, Yumi Okamoto, Shota Takada, Nobu Shirai, Tomomi Fujimoto, Genta Ochi, Koya Yamashiro, Daisuke Sato, Tatsuro Amano

**Affiliations:** 1 Laboratory for Exercise and Environmental Physiology, Faculty of Education, Niigata University, Niigata 950-2181, Japan; 2 Laboratory of Exercise Biochemistry and Neuroendocrinology, Faculty of Health and Sport Sciences, University of Tsukuba, Tsukuba City 305-8574, Japan; 3 Japan Society for the Promotion of Science, Tokyo 102-0083, Japan; 4 Department of Psychology, College of Contemporary Psychology, Rikkyo University, Saitama 352-8558, Japan; 5 Institute for Human Movement and Medical Sciences, Department of Health and Sports, Niigata University of Health and Welfare, Niigata 950-3198, Japan

**Keywords:** motor memory, skill training, global warming, heat loss response

## Abstract

This study aimed to determine whether heat exposure attenuates motor control performance and learning, and blunts cardiovascular and thermoregulatory responses to visuomotor accuracy tracking (VAT) tasks. Twenty-nine healthy young adults (22 males) were divided into two groups performing VAT tasks (5 trials × 10 blocks) in thermoneutral (NEUT: 25 °C, 45% RH, *n* = 14) and hot (HOT: 35 °C, 45% RH, *n* = 15) environments (acquisition phase). One block of the VAT task was repeated at 1, 2, and 4 h after the acquisition phase (retention phase). Heat exposure elevated skin temperature to ~3 °C with a marginally increased core body temperature. VAT performance (error distance of curve tracking) was more attenuated overall in HOT than in NEUT in the acquisition phase without improvement in magnitude alteration. Heat exposure did not affect VAT performance in the retention phase. The mean arterial blood pressure and heart rate, but not for sweating and cutaneous vascular responses to VAT acquisition trials, were more attenuated in HOT than in NEUT without any retention phase alternations. We conclude that skin temperature elevation exacerbates motor control performance and blunts cardiovascular response during the motor skill acquisition period. However, these alternations are not sustainable thereafter.

## 1. Introduction

Heat stress is well-recognized as impairing physical (e.g., endurance) exercise performance through thermoregulatory and cardiovascular function alternations [1,2]. Additionally, heat stress attenuates executive and cognitive functions especially for relatively complex tasks, such as working memory [3] and visual motor tracking [4], rather than easier ones (e.g., choice reaction time and memory recall) [5,6,7]. Studies indicate heat stress magnitude as a key factor for modulating executive and cognitive functions. For example, a high core body temperature (T_co_, ≥39.0 °C) is likely more related to attenuations in performance relative to those of lower T_co_ elevations [8]. Moreover, high-temperature environment (≥44 °C) exposure impairs executive function by increasing skin temperature (T_sk_) to ~3 °C without marked elevations in T_co_ [9,10]. Attenuations in complex cognitive performance by elevating T_sk_ are thought to be induced by alternations in the subjective state of thermal sensation and comfort [9,10].

Motor skill learning, which is defined as an increased spatial and temporal accuracy of movements with practice [11], is commonly involved in sports and industrial settings during hot summer environments. However, no study has yet investigated whether heat stress alters motor learning performance. Several studies demonstrated that repeating the visuomotor accuracy tracking (VAT) task rapidly improves the early stage performance of learning (i.e., fast learning), in which a response is modulated by cognitive processing [12,13]. This improved motor skill performance is consolidated as early as following several hours [14,15,16,17], modulated by the changes in primary motor cortex excitability [16,18]. Heat stress is assumed to alter motor learning of VAT tasks during the early stage of motor skill practice because heat stress affects central neural activities governing cognitive processing [19,20].

Autonomic nervous adjustments, including thermoregulatory and cardiovascular controls, play important roles in optimizing exercise in the heat. However, fewer studies have investigated the effects of heat stress combined with executive and cognitive tasks on thermoregulatory and cardiovascular responses, which could potentially lead to orthostatic intolerance [21]. Additionally, heat stress (T_co_, ~38.3 °C) blunts blood pressure response to sympathetic excitations induced by mental arithmetic [21] and cold pressor test [22]. Moreover, heart rate (HR), blood pressure, and sweating responses to isometric handgrip exercise are blunted at moderate (T_co_ elevation of ~0.6 °C) to high (~1.4 °C) heat stress conditions [23,24]. These blunted cardiovascular and thermoregulatory responses to various sympathetic stimuli under heat stress conditions may further deteriorate as the tasks are repeated. However, cardiovascular and thermoregulatory responses to repeated bouts of motor control tasks have yet to be examined under heat stress conditions.

The present study aimed to investigate the effect of heat exposure, which elevates mean skin temperature to >3 °C on motor control performance and learning in the VAT tasks and on cardiovascular and thermoregulatory responses during the VAT tasks in young healthy adults. We hypothesized that heat exposure would impair motor control performance during the early stage of learning, but it would not alter the maintenance of the improved performance compared to a non-heat stress condition. We also hypothesized that HR and blood pressure as well as sweating and cutaneous vascular responses during VAT tasks would be blunted by the heat exposure throughout the motor learning process.

## 2. Materials and Methods

### 2.1. Ethical Approval

The present study was approved by the human ethical committee of Niigata University (reference #2018-3-010). Verbal and written informed consent was obtained from all participants before the commencement of the experimental sessions.

### 2.2. Participants

The present study included 29 healthy right-handed young adults (22 males and 7 females). None of the participants had experienced VAT tasks before the participation. They did not take any prescription medications, and all were non-smokers. Participants were physically active students in Niigata University and were residents in Niigata, Japan. Participants were randomly assigned to one of the two groups performing VAT in hot (HOT, *n* = 15, 11 males) or thermoneutral (NEUT, *n* = 14, 11 males) environments. Age (20 ± 1 and 20 ± 1 years, *p* > 0.999), body mass (63.7 ± 9.3 and 64.3 ± 6.7 kg, *p* = 0.852), and height (170.5 ± 6.2 and 168.1 ± 9.0 cm, *p* = 0.435) did not differ between HOT and NEUT, respectively. All experimental trials were conducted between March and June 2019 to avoid the hot summer period (i.e., July to September) in Japan.

A minimum sample size of 28 (14 for each group) was required to detect the interactional effect between groups and trials on VAT performance with a power of 80% and an 𝛼-error probability of 0.05 based on an expected partial η^2^ of 0.06 (medium effect). Due to the lack of our (researchers in Niigata University) experience in VAT task and of previous studies assessing repeated VAT performance in the heat, it was difficult for us to determine a valid effect size prior to the study. Heat exposure with a high elevation of core temperatures attenuates cognitive motor performance with a large effect [4,25]. We thus estimated that the magnitude of heat-related attenuations in repeated VAT performance would be medium in our experiments, since we employed mild heat stress without a large increase in core temperature.

### 2.3. Experimental Protocol

**Figure 1:**
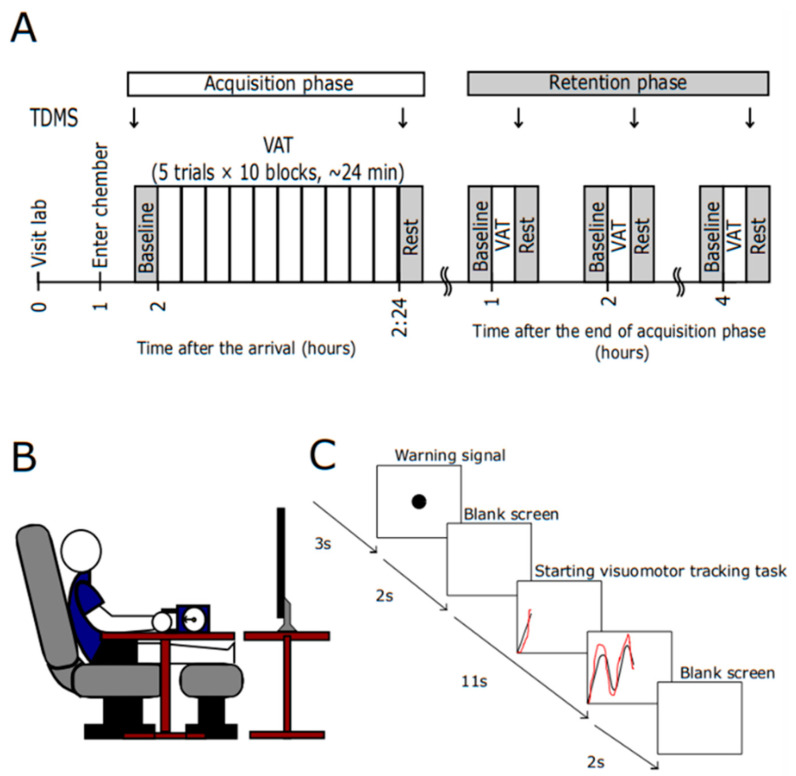
Experimental procedure (**A**), experimental setting (**B**), and a paradigm of a visuomotor accuracy tracking (VAT) trial (**C**) in the present study. TDMS: two-dimensional mood scale.

Figure 1A overviews the experimental protocol in the present study. Participants were instructed to refrain from consuming caffeine and alcohol and from participating in strenuous physical activity at least 24 h before the experimental trials, to consume 500 mL of water the night before each experiment, and to finish any meals and water (500 mL) at least 2 h before the experimental session. Participants reported to the laboratory at approximately 12:00 (±1 h). Upon arrival, urine samples were collected to assess hydration status, as determined by urine-specific gravity measurement. Euhydration was confirmed at a urine-specific gravity of ≤1.025 [26]. Body mass and height were then assessed using a weighing scale (DP-7800PW; Yamato Scale Co., Hyogo, Japan) and a stadiometer (YS501-P; Sanyu Co., Tokyo, Japan), respectively.

The participants were dressed in sport shorts and T-shirts, and the environmental chamber (FLC-15S: Fuji medical science) was regulated at 25 °C and 35 °C with 45% relative humidity for the NEUT and HOT conditions, respectively. They then rested in a sitting position (Figure 1B) for 1 h to habituate to the ambient conditions while the instrumentation was completed, during which maximum voluntary contraction (MVC) was assessed to determine their handgrip exercise intensity (<5% MVC) for the VAT trials. The participants performed 5 VAT trials for familiarization. Physiological responses and mood status (two-dimensional mood scale (TDMS)) [27] at baseline (BL) were assessed for 5 min after the habituation to the ambient conditions. Subsequently, the participants performed 10 blocks of the VAT task (5 trials per block) followed by a 5 min post-trial resting (defined as the acquisition phase).

Participants stayed at rest for an additional 4 h under the same environmental conditions (defined as the retention phase). They performed 1 block of VAT tasks at 1, 2, and 4 h after the end of the acquisition phase to evaluate the acquired motor control performance and physiological responses during VAT tasks. The participants were allowed to read books but not to sleep during the 4 h retention phase, as well as drink water ad libitum to prevent dehydration throughout the experiment. Body mass was assessed at the end of the experiments.

### 2.4. VAT Task

Participants performed the VAT task with their right hand using a hand dynamometer (GRIP-D, Takei scientific instruments, Niigata, Japan). The output signal was amplified and stored on a computer via a dated interface (AO-8CH, Applied Office Corporation, Tokyo, Japan). A software application (DASYlab version 2016, Measurement computing, Norton, MA, USA) was used to design the VAT task. A task window was displayed on a monitor (LCD-MF277XDB, I-O DATA, Ishikawa, Japan) placed in front of the participant (Figure 1B). One VAT trial takes 18 s. A warning signal appeared on the window for 3 s, followed by a blank screen display for 2 s. The window then showed seven sinusoidal curves from left to right of the monitor with a constant velocity (11 s). A red cursor, responding to a power output from the hand dynamometer, was also displayed on the same window moving from left to right with the same velocity of the target curve. The participants were instructed to trace the target sine waves as accurately as possible by controlling their grips to move the red cursor upward or downward using a dynamometer. The amplitude of the sine curve was adjected to 5% MVC. A blank screen was displayed for 2 s after the trial. The trial during the acquisition phase consisted of 10 blocks of VAT tasks (5 VATs per block) separated by a 1 min break between the blocks. The VAT task during the retention phase was 1 block.

### 2.5. Measurements

A thermistor probe (401J; Nikkiso-thermo, Tokyo, Japan) inserted 12 cm past the anal sphincter was used to measure the rectal temperature as an index of T_co_. Skin temperatures were measured by thermistors (ITP082-25; Nikkiso-thermo, Tokyo, Japan) affixed to four skin sites. The mean skin temperature (T_sk_) was calculated using the four skin temperatures as follows [28]: chest, 30%; upper arm, 30%; thigh, 20%; and lower leg, 20%. A data storage device (model N543; Nikkiso-thermo, Tokyo, Japan) was used to record the rectal and skin temperatures at 1 s intervals.

HR and mean arterial blood pressure (MAP) were continuously measured using a Finometer device (Finapres Medical Systems, Amsterdam, The Netherlands). The ventilated capsule method was used to continuously measure the local chest and palm sweat rate (SR). A sweat capsule was attached to the palm of the other hand not used for the exercise. A 5.30 cm^2^ plastic capsule was secured to the skin using topical glue (Collodion; Kanto Chemical, Tokyo, Japan). Dry nitrogen gas was ventilated through the capsule at a flow rate of 1.0 L min^−1^. A capacitance hygrometer (HMP60; Vaisala, Helsinki, Finland) was used to measure the water content from the effluent air. Chest and left palm skin blood flows were continuously measured using a laser-Doppler velocimetry (FLO-C1; Omegawave, Tokyo, Japan). A laser-Doppler probe was located adjacent to the ventilated capsule. Cutaneous vascular conductance (CVC) was calculated from the ratio of skin blood flow to MAP. SR and skin blood flow were recorded at 1 s intervals using a data logger system (MX100; Yokogawa, Tokyo, Japan).

Comfort and thermal sensations were assessed at the breaks between blocks using a Gagge scale [29] ranging from 1 to 4 as comfortable to very uncomfortable and from 1 to 8 as cold to hot, respectively. TDMS was used to assess the psychological mood status to evaluate arousal levels based on the manufacturer’s guidance.

VAT performance was determined as the average root mean square of an error distance between the performed power signals produced by handgrip exercise and displayed target sine waves across all sampled data points (1000 Hz) in each frame.

### 2.6. Data and Statistical Analysis

All continuously measured variables were averaged for 5 min at BL and post-VAT recovery period. These measurements were averaged every block during the acquisition and retention phases. Changes in physiological responses to the VAT trials from BL before each VAT trial were calculated and used for the statistical analysis, except for T_co_ and T_sk_. Relative changes in VAT performance (root mean square of an error distance) from blocks 1 and 10 for the acquisition and retention phases were calculated, respectively.

A two-way repeated measures analysis of variance (ANOVA) for VAT performance and physiological responses during the acquisition phase was performed as the repeated (10 or 12 levels; 10 blocks with or without BL and post-VAT recovery) and non-repeated factor of block groups (2 levels: NEUT and HOT). A two-way repeated measures ANOVA was used to evaluate the VAT performance and physiological responses during the retention phase as the repeated factors of protocol stages (3 levels: 1, 2, and 4 h after the acquisition phase) and non-repeated factors of group. The Greenhouse–Geisser correction was applied if the assumption of sphericity was violated. A Q-Q plot assessment was used to confirm a normal distribution of variance. A post hoc analysis was performed using Bonferroni’s multiple comparison test. Some variables were analyzed in reduced participants due to measurement errors, which were identified in the figure legends or tables. Data are presented as means ± standard deviation (SD), and statistical significance was set at ≤0.05. All statistical analyses were performed using Prism (version 8.1.2, GraphPad Software, San Diego, CA, USA).

## 3. Results

### 3.1. VAT Task Performance

**Figure 2:**
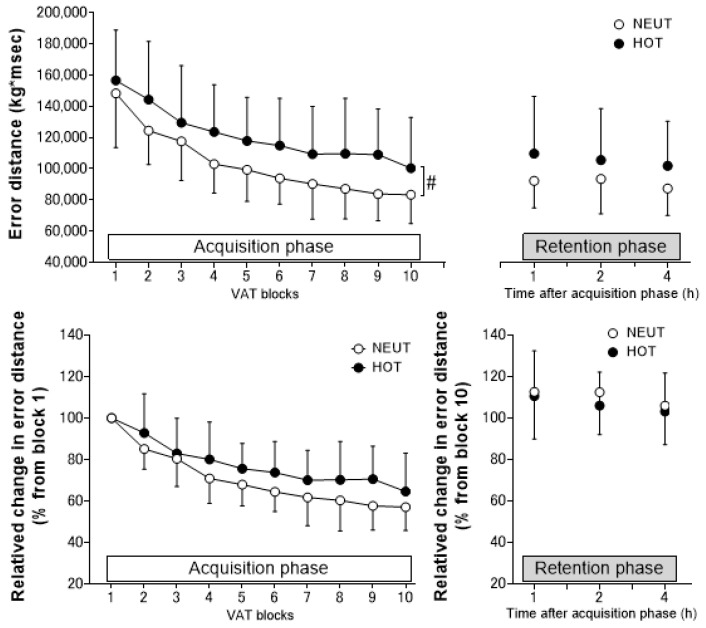
Absolute and relative changes in error distance during the acquisition and retention phases in thermoneutral (NEUT, *n* = 14) and hot (HOT, *n* = 15) environments. Values are presented as means ± SD. #, the main effect of environmental condition (*p* = 0.050).

The error distance of the VAT task during the acquisition phase was higher in HOT compared to NEUT (main effect of environment, *p* = 0.050) but without significant interaction effects (*p* = 0.585, Figure 2). The relative changes from block 1 in error distance of the VAT task did not differ between HOT and NEUT during the acquisition phase (*p* = 0.078 and *p* = 0.236 for the main effect of environment and interaction, respectively). Heat exposure did not affect the absolute (*p* ≥ 0.120 and *p* = 0.757 for the main effect of environment and interaction, respectively) and relative (percentage of block 10) (*p* ≥ 0.530 and *p* = 0.825, respectively) changes in error distance of the VAT task compared to the thermoneutral trial during the retention phase.

### 3.2. Core Body and Skin Temperatures

**Figure 3:**
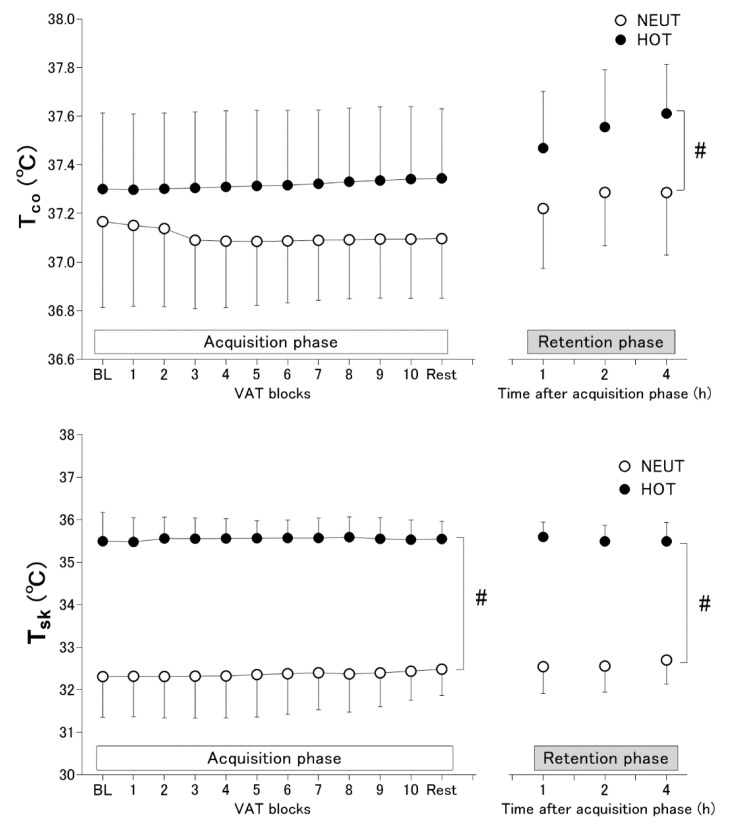
Core body (T_co_) and mean skin (T_sk_) temperatures during VAT tasks in thermoneutral (NEUT, *n* = 14) and hot (HOT, *n* = 15) environments for acquisition (left panel) and retention (right panel) phases. Values are presented as means ± SD. #, the main effect of environmental condition (all *p* ≤ 0.003).

T_co_ did not differ between HOT and NEUT in the acquisition phase (*p* = 0.060). A significant interaction effect of the environment and protocol stage was observed in T_co_ in the acquisition phase but without differences in any VAT blocks (all *p* ≥ 0.242) (Figure 3). Conversely, T_co_ was more elevated in HOT than in NEUT in the retention phase (main effect of environment, *p* = 0.003). Heat exposure elevated T_sk_ in both acquisition and retention phases (both *p* < 0.001 for the main effect of the environment) (Figure 3).

### 3.3. Cardiovascular Responses

**Figure 4:**
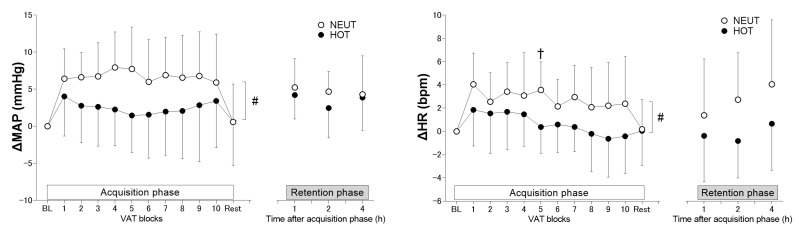
Changes from baseline (BL) before each VAT trial in mean arterial pressure (ΔMAP) and heart rate (ΔHR) during VAT tasks in thermoneutral (NEUT, *n* = 13) and hot (HOT, *n* = 15) environments for acquisition (left panel) and retention (right panel) phases. Values are presented as means ± SD. #, the main effect of environmental condition, all *p* ≤ 0.038. †, NEUT vs. HOT at each block (*p* = 0.017).

The resting MAP before each VAT tasks were not different between environmental conditions but was higher in HOT than in NEUT in HR (*p* = 0.024, the main effect of environment, Table 1). ΔMAP and ΔHR during the VAT trial in the acquisition phase were more attenuated in HOT than in NEUT conditions (main effect of environment, *p* = 0.031 and *p* = 0.038, respectively). An environment × group interaction was observed for both ΔMAP and ΔHR (*p* = 0.012 and *p* = 0.021, respectively), but the post hoc analysis only reveals a difference in HR at the 5th VAT blocks (*p* = 0.017, Figure 4). Environmental condition did not affect ΔMAP and ΔHR during VAT trials between NEUT and HOT conditions in the retention phase (all *p* ≥ 0.308 and *p =* 0.539 for the main effect of environment and interaction, respectively) (Figure 4).

### 3.4. Thermoregulatory Responses

**Figure 5:**
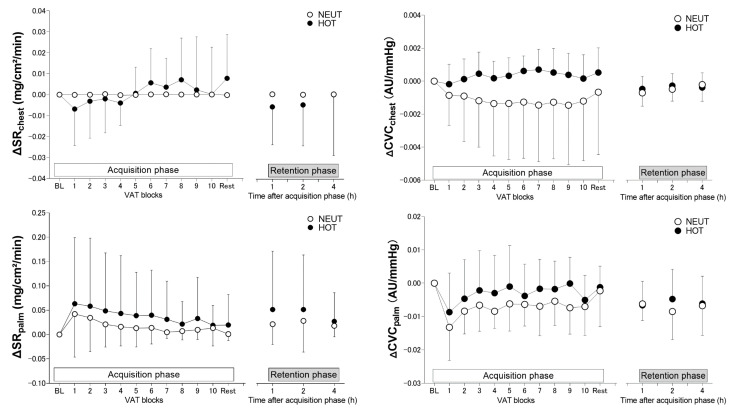
Changes from baseline (BL) before each VAT trial in sweat rate on the chest (ΔSR_chest_, *n* = 14 and *n* = 15 for the NEUT and HOT conditions, respectively) and palm (ΔSR_palm_, *n* = 14 and *n* = 15, respectively), as well as cutaneous vascular conductance on the chest (ΔCVC_chest_, *n* = 13 and *n* = 15, respectively) and palm (ΔCVC_palm_, *n* = 12 and *n* = 15, respectively), during VAT tasks in thermoneutral (NEUT, *n* = 13) and hot (HOT, *n* = 15) environments for acquisition (left panel) and retention (right panel) phases. Values are presented as means ± SD.

Participants sweat in the HOT but not NEUT conditions throughout the experiment (Table 1). This results in higher resting SR on the palm and chest, as well as CVC on the palm but not chest before each VAT task in HOT as compared to NEUT (all *p* ≤ 0.028 for the main effect of the environment). ΔSR on the chest and palm as well as ΔCVC on the palm did not differ between the HOT and NEUT conditions in either acquisition (all *p* ≥ 0.753 and *p* = 0.942 for the main effect of environment and interaction, respectively) and retention (all *p* ≥ 0.603 and *p* = 0.648, respectively) phases, respectively (Figure 5). A significant interaction effect (*p* = 0.015) but not main effect of environment (*p* = 0.082) was observed in ΔCVC on the chest during the VAT trial in the acquisition phase but without a time-specific difference (all *p* ≥ 0.591). Chest ΔCVC during the VAT trial in the retention phase did not differ between the environmental conditions (*p* = 0.607 and *p* = 0.523 for the main effect of environment and interaction, respectively) (Figure 5).

### 3.5. Subjective Variables

**Figure 6:**
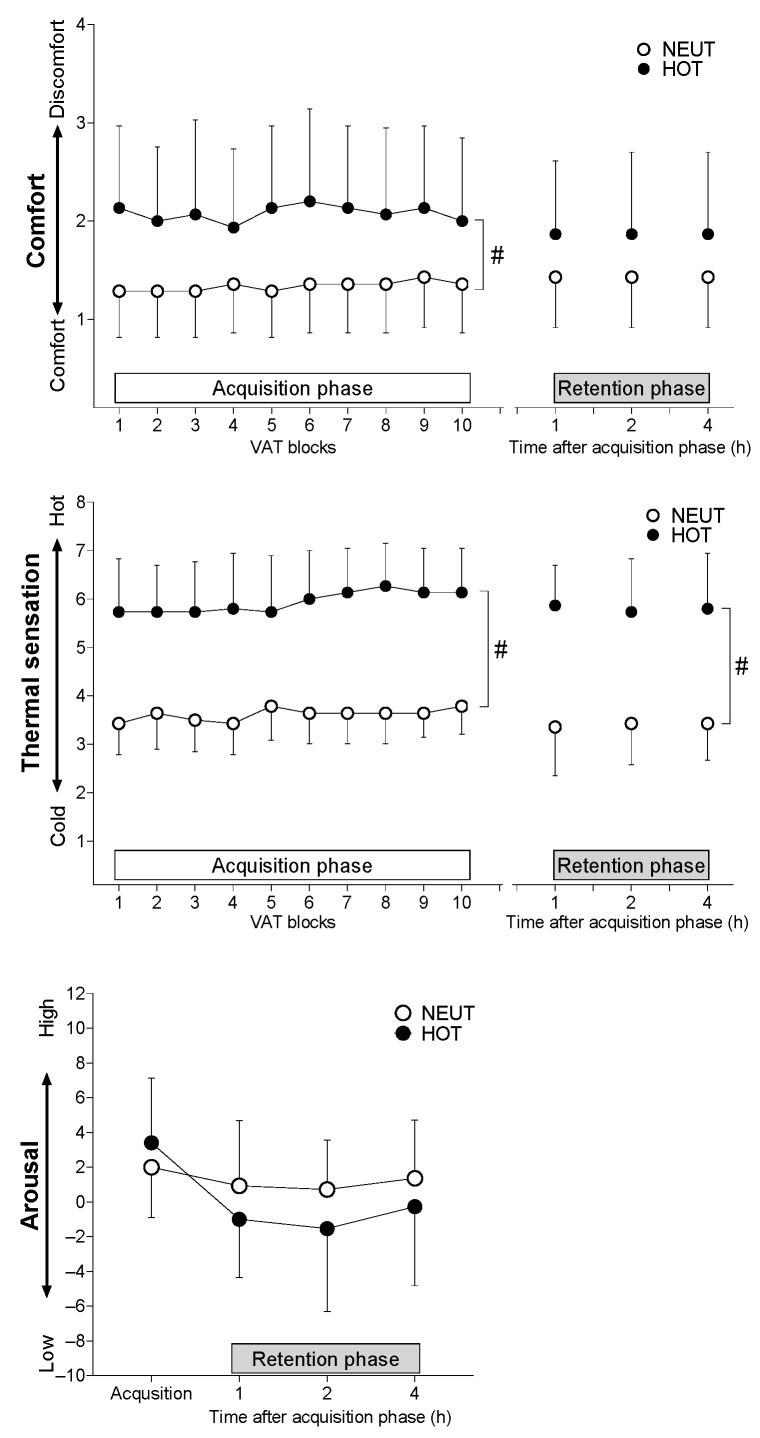
Comfort and thermal sensations during VAT tasks in thermoneutral (NEUT) and hot (HOT) environments for acquisition (left panel) and retention (right panel) phases. Values are presented as means ± SD. *n* = 14 and 15 for NEUT and HOT conditions, respectively. #, the main effect of environment (all *p* ≤ 0.005).

Heat exposure increased discomfort (*p* = 0.005 for the main effect of the environment) and thermal (*p* < 0.001) sensations during VAT trials compared to those of NEUT in the acquisition phase but without significant interaction effect in both sensations (both *p* ≤ 0.529) (Figure 6). Thermal sensation remained elevated in the HOT condition compared to the NEUT conditions in the retention phase (*p* < 0.001 and *p* = 0.768 for the main effect of environment and interaction, respectively), without a difference in comfort sensations between the environmental conditions (*p* = 0.077 and *p* > 0.999, respectively) (Figure 6).

### 3.6. Other Variables

Urine-specific gravity assessed upon arriving at the laboratory did not differ between the HOT and NEUT conditions (*p* = 0.144, 1.016 ± 0.006 and 1.021 ± 0.010, respectively). Fluid consumption during the experiment was greater in HOT as compared to NEUT (*p* < 0.001, 229 ± 133 and 64 ± 84 g, respectively). Whole body sweat loss throughout the experiment adjusted to the weight of ingested fluid was greater in the HOT condition as compared to the NEUT condition (*p* < 0.001, −0.7 ± 0.2 and −0.3 ± 0.1 kg, respectively).

## 4. Discussion

HOT increased T_sk_ to ~3 °C and T_co_ to ~0.4 °C compared to the NEUT condition throughout the experiments (~5 h). This systemic and long-lasting heat stress attenuates motor control VAT performance during the acquisition phase. However, the heat exposure-induced performance impairment disappeared at least 1 h after the acquisition trial. Second, the changes in MAP and HR but not sweating and cutaneous vascular responses to VAT trials throughout skill acquisition were attenuated by the heat exposure. However, the blunted cardiovascular responses to the VAT trial disappeared when performed 1–4 h after the acquisition phase. Third, heat exposure increased the thermal sensation and discomfort throughout skill acquisition. In parallel with the performance responses, the discomfort was reduced at least 1 h after the end of the skill acquisition trial while thermal sensation remained elevated in the HOT compared to NEUT conditions. These results suggest that a large increase in skin temperature with marginal elevation in core temperature impairs motor control performance and cardiovascular adjustments over repeated bouts of motor control trials. These impairments did not sustain even for 1 h and up to 4 h after the trial despite a maintained heat exposure. Changes in discomfort may in part be responsible for the altered performance response to VAT trials in a hot environment.

### 4.1. Effects of Heat Exposure on VAT Performance and Learning

We have demonstrated that heat exposure to 35 °C hot air increases skin temperature to ~3 °C and attenuates VAT performance over the skill acquisition process. The attenuation of VAT performance during the acquisition phase in heat was likely induced by skin temperature elevation because T_co_ elevation was not pronounced in the HOT conditions (Figure 3). This is consistent with previous studies demonstrating that a high skin temperature alters complex cognitive performance [10]. Heat exposure is considered to attenuate overall response in motor control performance throughout the skill learning process (acquisition phase) without altering the magnitude of improvement because the relative changes in skill performance from block 1 did not differ between HOT and NEUT conditions in the acquisition phase (Figure 2). Assumingly, the magnitude of physiological heat stress (i.e., T_co_ elevation) might not be enough to alter the magnitude of improvement in VAT performance in the present study.

The precise mechanism(s) for attenuating heat exposure-induced VAT performance is unknown. However, consistent with previous studies reported in complex cognitive tasks [4,7,10], alternations in subjective feelings by heat exposure, such as thermal sensation and discomfort, may play a role in attenuating VAT performance. Interestingly, participants reported less discomfort in the heat similar to the level in the NEUT environment at least 1 h after the VAT acquisition cessation, in parallel with the absence of VAT performance difference at the retention phase. Thermal sensation remained elevated until the end of the heat exposure. Thus, we assumed discomfort to be a feeling that would increase cognitive load [30] rather than thermal perception to be responsible for VAT performance attenuation in the heat. Heat stress is suggested to modulate regional brain blood flow and to thus potentially alter cognitive function [7,8]. Conversely, a recent study demonstrated that neither skin temperature elevation nor end-tidal carbon dioxide or cerebral blood flow modulates cognitive function during hyperthermia [31]. Therefore, future research is required to determine a potential link between VAT performance and brain blood flow in the heat.

VAT performance did not differ 1–4 h after the end of acquisition phase (Figure 2). This is probably due to the changes in discomfort returning to a level similar to the NEUT environment despite continued heat exposure. Assumingly, participants were familiarized with the hot environment during a stay in the hot chamber and felt less discomfort in modulating VAT performance. Consistent with our hypothesis, heat exposure did not affect relative VAT performance changes in the retention phase from block 10 of the acquisition phase. Excitability of the primary motor cortex rather than cognitive processing is believed to modulate motor skill consolidation after the phase of the first learning (e.g., maintenance of performance after acute motor skill learning) [32]. Therefore, we speculate that increased skin temperature by +3 °C by heat exposure may not influence primary motor cortex excitability while future studies warrant further investigation.

### 4.2. Effects of Heat Exposure on Cardiovascular and Thermoregulatory Responses to the VAT Trial

Previous studies demonstrated that cardiovascular and thermoregulatory responses to acute physiological stimuli, such as mental arithmetic [21], cold pressor test [22], and handgrip exercise [23], were attenuated in the heat, accompanied by an increased core temperature of at least to 0.6 °C. The VAT task employed in the present study elicited limited elevations in MAP (~10 mmHg) and HR (~3 bpm) in the NEUT condition compared to those of previous studies (e.g., ~30 mmHg in MAP and ~30 bpm in HR) [21,22,23], probably due to the weak exercise intensity employed (5% MVC). Therefore, our results suggest that heat stress attenuates cardiovascular responses to VAT tasks in the acquisition phase even with a weak physiological stimulus and an inadequate marked core temperature elevation. A high skin temperature is a key modulator blunting cardiovascular responses to the VAT task in the acquisition phase.

Overall MAP and HR responses were attenuated by heat exposure throughout the motor skill acquisition trial (i.e., the main effect of the environment). No significant differences were found in SR on the palm, which is a skin region sensitive to autonomic sympathetic activations, between hot and thermoneutral environmental conditions. Therefore, the attenuations in cardiovascular responses to VAT tasks by heat exposure might not be related to a potential alternation in efferent sympathetic activity. Heat stress does not modulate muscle sympathetic nerve activation to cold pressor test [22], or it does increase the response to mental stress [21] despite reductions in blood pressure responses. Blunted increases in cardiac output or vascular resistance may contribute to a MAP reduction during handgrip exercise [23] or cold pressor test [22] in the heat. We did not evaluate cardiac output and vascular resistance in the present study, but chest CVC was higher in HOT than in NEUT environments (Figure 5). This implies a possibility that peripheral vascular-related mechanisms might contribute to the attenuated blood pressure response to VAT trial in the acquisition phase.

MAP and HR responses to VAT were not different between the HOT and NEUT conditions evaluated at 1–4 h after the motor skill acquisition cessation (i.e., retention phase) despite maintaining elevated skin temperature. The precise reason(s) for the abolished effects of heat exposure on cardiovascular responses to VAT tasks during the retention phase is unknown. However, only one block of the VAT task was performed during the retention phase, but it was repeated 10 times in the acquisition phase. Therefore, we could not exclude the possibility that one block of the VAT task was not adequate to evaluate cardiovascular responses during the retention phase because the VAT task did not induce large physiological responses. Further research is required to elucidate whether a long-lasting heat exposure for several hours does not modulate cardiovascular responses to motor control tasks by employing perturbations inducing large physiological responses.

### 4.3. Perspectives and Significance

Climate change is a crucial factor modulating human health and performance. An increase in environmental temperature increases the opportunity to work and exercise in hot environments. The present study provides important implications for individuals repeating motor control tasks in hot environment including sports (e.g., exercise skill training), occupational settings (e.g., firefighter), and military trainings. It would be important for these individuals to recognize that the performance (or ability) of repeated motor tasks may be attenuated in the heat. However, further studies are required to develop optimal intervention(s) to prevent reductions in performance and cardiovascular response during repeated bouts of motor control task in the heat.

### 4.4. Limitations

There are several limitations in the present study. First, all experimental trials were performed starting at around noon. We are therefore uncertain how the outcomes could be influenced by the circadian cycle when the study is conducted at different time periods. Second, as we recruited young healthy adults only, we cannot expand the results to other populations such as children and older adults. Further studies are required to elucidate potential differences in the effect of heat exposure on repeated VAT task performance and cardiovascular as well as thermoregulatory responses in different populations, including sex differences. Third, we assessed thermal stress from rectal and skin temperatures obtained from four skin sites, all of which are well accepted measures in thermal physiology. However, as the index of core and skin temperatures could differ between the site of assessments [33], it is noted that the magnitude of thermal stress in the present study is limited in our body temperature assessments only. Fourth, in keeping with previous study protocols [14,15,34], we employed a between-subjects design rather than a within-subjects design to avoid potential effects of previous experience in VAT tasks [34]. Future research is required employing within-subjects design with a valid washout period to obtain robust outcomes. Finally, it remains unknown if and how participant’s experiences in regular motor control tasks could potentially affect the outcome in the present study.

## 5. Conclusions

In conclusion, the present study demonstrated for the first time that heat exposure to increase skin temperature exacerbates motor control performance assessed by the VAT task throughout the motor skill acquisition process (repeated bouts). However, heat exposure does not exacerbate the retention of acquired VAT performance. Cardiovascular responses to repeated VAT tasks during the motor skill learning process were attenuated by the heat exposure, while this attenuation is abolished as early as 1 h after skill acquisition cessation. Heat-related discomfort might play a role in modulating VAT performance in the heat.

## Figures and Tables

**Table 1:** Physiological variables at rest before VAT task.

			Acquisition Phase	Retention Phase (Time after Acquisition Phase, h)		
				1	2	4
MAP (mmHg)		NEUT	85.9 ± 6.0	86.3 ± 9.3	83.6 ± 9.7	89.0 ± 9.8
		HOT	82.7 ± 5.6	85.0 ± 11.8	88.3 ± 11.4	87.2 ± 12.3
HR (bpm)	#	NEUT	65.4 ± 7.5	64.3 ± 7.4	63.7 ± 7.1	65.3 ± 8.1
		HOT	71.0 ± 7.4	71.2 ± 8.5	72.5 ± 9.8	71.8 ± 7.5
SR_chest_ (mg/cm^2^/min)	#	NEUT	0.01 ± 0.00	0.01 ± 0.00	0.01 ± 0.00	0.01 ± 0.00
		HOT	0.08 ± 0.05	0.09 ± 0.06	0.10 ± 0.06	0.10 ± 0.06
SR_palm_ (mg/cm^2^/min)	#	NEUT	0.04 ± 0.03	0.04 ± 0.04	0.05 ± 0.05	0.04 ± 0.04
		HOT	0.14 ± 0.03	0.12 ± 0.09	0.14 ± 0.14	0.16 ± 0.22
CVC_chest_ (AU/mmHg)		NEUT	0.011 ± 0.005	0.010 ± 0.003	0.010 ± 0.003	0.010 ± 0.003
		HOT	0.012 ± 0.004	0.012 ± 0.005	0.012 ± 0.004	0.013 ± 0.005
CVC_palm_ (AU/mmHg)	#	NEUT	0.026 ± 0.012	0.022 ± 0.010	0.022 ± 0.007	0.017 ± 0.009
		HOT	0.004 ± 0.013	0.050 ± 0.013	0.046 ± 0.011	0.045 ± 0.014

Values are presented as means ± SD. ΔSR_chest_ and ΔSR_palm_, sweat rate on the chest and palm (*n* = 14 and *n* = 15 for NEUT and HOT conditions, respectively); ΔCVC_chest_ and ΔCVC_palm_, cutaneous vascular conductance on the chest (*n* = 13 and *n* = 15, respectively) and palm (*n* = 12 and *n* = 15, respectively). #, the main effect of environment (all *p* ≤ 0.028).

## Data Availability

The data that support the findings of this study are available from the corresponding author upon reasonable request.
